# Free Trade in Medicine and Surgery

**Published:** 1847-02

**Authors:** 


*Free trade in Medicine and Surgery.*—The people called "statesmen" as a grove is called *lucus, a non lucendo*, have taken into their wise noddles the strange crotchet that every man, woman, and child in England, inherits and enjoys the unquestionable right to cripple, poison, mutilate, and slay all and every her Majesty's subjects, provided they consider that they can thereby put money in their purses, and all dyspeptic lawyers and constipated jurymen have evidently arrived at the same conclusion. Mr. Warburton, the great theoretical medical reformer, by his advocacy of cheap-doctoring and free trade in drugs, did much to perpetuate this delusion, and Sir James Graham by his abortive bill contributed to the same end. Every day's experience, however, proves that there must be an end to this nonsense. The recorded deaths from the mal-administration of drugs form a frightful catalogue of wanton outrages within the present year, and the evil every day increases. The following is another example of the toleration extended to the base and ignorant who have the temerity to trifle with life in this way. Judges, lawyers, and jurors have become so familiarized with such displays that they have now become subjects of jest, or occasions for cracking jokes on the doctors.—*Dublin Med. Press.*

"A report having been circulated that a man named William Myhill, a small farmer and carpenter, residing at Horsey, in the county of Norfolk, had died from the effects of some medicine which had been administered to him by his wife, Mr. Pilgrim, the county coroner, directed the body to be exhumed, and on the 24th of last month held an inquest at Catfield, where the body had been interred. Several witnesses were examined, but the chief evidence offered was that of the servantmaid, who, in a long statement, disposed to her mistress having obtained some medicine of a person living at Reepham, which she administered to the deceased just previous to his death, and then requested her (the servant,) not to say anything about it to any person, but to deny it if she was asked any questions on the subject. On Friday, October 2nd, the inquiry was resumed, when amongst other witnesses who were examined as to the wife having administered something to the deceased, was a Mr. Staples of Reepham, who calls himself a chemist and druggist. He deposed as follows:—

"I vend drugs and prepare them, but I do not profess to be a surgeon. Some short time since Mrs. Myhill, the wife of the deceased, came to me and stated that her husband was very bad. I prescribed for the deceased from the representation made to me by his wife. I cannot say what she stated. I made up some medicine according to the nature of the disease. I was not told what was the matter with him, but I found it out by my study, my science, and my search. I do not recollect that I ordered brandy and water, neither do I exactly recollect what I did prescribe. At the time I put it on a slate, but it was afterwards rubbed off. The medicine was to relieve the pain—it was not opening medicine. Mrs. Myhill was to have called upon me again, and let me know how her husband was, and to tell me the effect the medicine had upon him. I am perfectly satisfied that the medicine I prescribed could not do him any harm, but I did intend that it should do him good. I considered that the deceased was in a very bad state, and that I ascertained from my research in science, and study from my books of knowledge. If a person came to me and represented their case, I should not be governed by what he said, but I should be governed by the rule of science and my books of knowledge. I could by searching those books ascertain more of their disease than any person could inform me. It is a very common practice with me to prescribe for persons I have never seen, nor yet had a description of their complaints. I neither want to know the name of the party, or where they come from, or any description whatever of their complaints, as I can always find every thing out by the rule of science, my study, and from my books of knowledge. If any person had come to me after the death of Myhill, I could have stated the cause of his death, but the time is now so far gone that I cannot. He again repeated his powers of discovering the complaints of persons by the aid of his books, which was the cause of much merriment to the coroner and the jury, who looked with some suspicion upon the many *cases* [cures?] he pretended to have effected by his books, his science, and his study.

After this evidence, which put a very different aspect upon the inquiry, the surgeons, who had analysed the stomach, said that they had not been able to detect the presence of any metallic or vegetable poison; and, from the appearance of the lungs, were of opinion that the deceased died from natural causes. The jury returned a verdict accordingly."—*Norwich Mercury.*

